# Gender, childhood and adult socioeconomic inequalities in functional disability among Chinese older adults

**DOI:** 10.1186/s12939-017-0662-3

**Published:** 2017-09-02

**Authors:** Yaqin Zhong, Jian Wang, Stephen Nicholas

**Affiliations:** 1 School of Public Health, Nantong University, 9 Seyuan Road, Nantong, 210029 Jiangsu People’s Republic of China; 2 School of Public Health, Shandong University, 44 Wenhuaxi Road, Jinan, 250012 Shandong People’s Republic of China; 3 School of Management and School of Commerce, Tianjin Normal University, West Bin Shui Avenue, Tianjin, 300074 People’s Republic of China; 4 Guangdong Research Institute for International Strategies, Guangdong University of Foreign Studies, 2 Baiyun North Avenue, Baiyun, Guangzhou, Guangdong, 510420 People’s Republic of China; 5 School of International Business, Beijing Foreign Studies University, 19 North Xisanhuan Avenue, Haidian, Beijing, 100089 People’s Republic of China; 6 University of Newcastle, Newcastle, NSW 2308 Australia

**Keywords:** Gender, Socioeconomic status, Inequality, Functional disability

## Abstract

**Background:**

Gender difference and life-course socioeconomic inequalities in functional disability may exist among older adults. However, the association is less well understood among Chinese older population. The objective is to provide empirical evidences on this issue by exploring the association between gender, childhood and adult socioeconomic inequalities in functional disability.

**Methods:**

Data from the 2013 wave of the China Health and Retirement Longitudinal Study (CHARLS) was utilized. Functional disability was assessed by the activities of daily living (ADL) and instrumental activities of daily living (IADL). Childhood socioeconomic status (SES) was measured by birthplace, father’s education and occupation. Adult SES was measured in terms of education and household income. Multivariate logistic regressions were conducted to assess the association between gender, childhood and adult SES and functional disability.

**Results:**

Based on a sample of 18,448 older adults aged 45 years old and above, our results showed that the prevalence of ADL and IADL disability was higher among women than men, but gender difference disappeared after adult SES and adult health were controlled. Harsh conditions during childhood were associated with functional disability but in multivariate analyses only father’s education was associated with IADL disability (OR for no education = 1.198; 95% CI = 1.062–1.353). Current SES such as higher education and good economic situation are protective factors of functional disability.

**Conclusions:**

Childhood and adult SES were both related to functional disability among older adults. Our findings highlight the need for policies and programs aimed at decreasing social inequalities during childhood and early adulthood, which could reduce socioeconomic inequalities in functional disability in later life.

## Background

Globally, 650 million people suffer from a disability, with an estimated 85 million people in China suffering from a disability that negatively affects their daily lives [1]. Disabilities are correlated with age. As China’s economic transition accelerates, China faces a rapidly aging population. It is estimated that the proportion of people over the age of 60 will increase from 15% in 2015 to 30% in 2050 of the Chinese population [2]; the ratio of working to non-working population will fall from 2.5 to 1.5 in 2050; and the current male-female gender imbalance will result in 15% more females aged over 45 years than males by 2050 [3]. The most common problem affecting the health and quality of life of older adults is functional disability. Functional disabilities impair a person’s activities due to physical or mental limitations related to illness, mobility handicaps and cognitive and sensory limitations compared to the normal activities of people without the disability [4]. The most widely used measurements of functional disability are the basic activities of daily living (ADL), instrumental activities of daily living (IADL) and mobility [4]. ADL are routine activities that people do every day without needing assistance, such as personal care and hygiene, mobility and eating, while IADL are higher level activities related to independent living in the community, such as meal preparation, shopping, housekeeping and managing money [5, 6]. Mobility refers to tasks related to individuals’ locomotor system. With China’s aging population, functional disabilities not only challenge the quality of live of millions of individuals, but also impose heavy social burdens through long-term care and increased levels of medical services [4].

Previous studies have described a range of risk factors for functional disability, including advancing age, gender differences, lack of schooling, and chronic diseases. But the results have been inconsistent. In regard to gender differences, some results revealed that women had higher prevalence of functional disability [7, 8], while other studies identified men as having higher risks [9] or showed no gender differences [10]. Yong found Singaporean women had more functional disabilities than Singaporean men [8], while Grundy and Glaser found higher incidence of functional disability in British men [9]. For elderly Brazilians, different comorbidity and susceptibility to socioeconomic and health exposures between women and men helped explain the disability gender gap [11]. Reviewing 21 cohort studies, Tas found the incidence of functional disability was similar between genders [10]. Since these gender studies vary in geographic coverage and sample size, it is hard to draw generalizable conclusions for China.

Studies have demonstrated that socioeconomic status (SES) variables, often measured by education, occupation and income, were strongly associated with functional disability [12–14]. In general, individuals with low SES were more vulnerable to the incidence of functional disability. Low SES was closely linked with limited access to medical services, inadequate nutrition and unhealthy behaviors that increased the prevalence of functional disabilities. Several SES studies have been conducted in China. Liu concluded that less education, rural residence, unemployment and lower income were associated with functional disability in Chinese older adults [15]. Also studying older Chinese, Hu drew similar conclusions [16].

Besides individuals’ current SES, there is increasingly evidence that, a child’s SES is an important factor explaining adult functional disabilities. The life-course perspective emphasizes that personal development is a lifelong process and childhood SES may have long term effects on adult health outcomes [17, 18]. The life course perspective offers various interpretations of how childhood SES may impact later health, including the biological programming model, the pathway model and the accumulation model [19]. The biological programming model argues that risks experienced in childhood that are then embedded in the internal structure and functioning of biological systems may have long-term effects on adult health, independent of later exposures [20]. In contrast, the pathway model emphasizes that early SES conditions lead to similar exposures in later life and alter life course trajectories. In effect, low childhood SES limits and shapes adult SES, with negative impacts on adult health. The cumulation model highlights the role of the accumulation of risk factors across the entire life course in determining ultimate health outcomes [21, 22]. Poor childhood SES conditions may initiate unhealthy behaviors, which continue into adulthood, increasing the risk of functional disability [23]. In short, childhood disadvantage may negatively determine later life trajectories and adult health including functional disabilities in later life.

Most studies focusing on gender and life-course socioeconomic inequalities have been conducted in western countries, which limits the extrapolation of their findings to developing countries [4, 24]. Several articles explored the association between childhood conditions and adult health in China [25–27]. Zeng [25] explored the association of childhood socioeconomic conditions with healthy longevity at the older ages and Ming Wen [26] investigated the effects of childhood SES on health and mortality among older Chinese. Zhang [27] explored early life influences on cognitive impairment among older Chinese. These studies focused on adults aged 65 and older, which limits the exploration of a younger age group and they did not discuss gender difference.

Our study explores whether functional disability varies according to gender and life course socioeconomic status in Chinese older adults. Our hypotheses are that gender differences exist in functional disability and low childhood and adult SES increase the risk of functional disability in later life through a variety of disadvantaged conditions and health behavioral factors. Second, we suggest preventive measures for functional disabilities and make recommendations for the organization of health services.

## Methods

### Data

Our data came from the 2013 wave of the China Health and Retirement Longitudinal Study (CHARLS), which studies health outcomes and economic adjustments to China’s rapid aging population. A national representative survey of China’s middle aged and elderly population, CHARLS comprises a three stage stratified probability proportionate to size (PPS) sample. CHARLS selected 10,803 households with individuals aged 45 and older from 450 villages or communities in 150 counties or districts of 28 provinces in China. While CHARLS targeted respondents aged 45 years old and above and their spouses at whatever age, we excluded all respondents younger than 45. Our study comprised 18,488 individuals aged 45 and older from 10,713 households. All analyses were weighed using individual sample weights, adjusted for non-response of household and individual. With its large sample size and high response rate [28], CHARLS offers an opportunity to examine older adults’ functional disability using a far larger sample than typical in such research.

### Variables and instruments

*Functional disability* was assessed by ADL and IADL [5, 6]. For ADL, respondents were asked to answer whether they had difficulty in performing six activities: dressing, bathing, eating, toileting, getting in or out of bed and controlling urination and defecation. The Cronbach’s alpha of these six items was 0.843, indicating good reliability and consistency. Answers were categorized as: have no difficulty; have difficulty but can still do it; have difficulty and need help; can not do it. For the purpose of multivariate analysis, we followed previous studies by using the dichotomized variable, no difficulty and one or more difficulties [29]. For IADL, respondents were asked whether they had difficulty in doing household chores, preparing meals, shopping, making calls, taking medications and managing money (Cronbach’s alpha = 0.802). A dichotomized variable was also constructed, so ADL/IADL disability was defined as having difficulty in one or more ADL/IADL items.

*Childhood socioeconomic status* was indicated through the following measures: urban-rural birthplace, father’s education and father’s occupation. For respondents born before 1970s, there were significant differences in health, working conditions and opportunities between rural and urban areas. Father’s education was classified as received some education or no education, which reflected the fact that few fathers of the respondents had the opportunity to attend school in the early twentieth century. Father’s occupations were dichotomized into non-manual (such as managers, professionals, technicians and clerks) or manual (including agricultural, forestry, husbandry and fishery producers) categories. The above indicators have been well validated and frequently used for classifying childhood SES for Chinese respondents in the previous studies [25, 26].

*Adult socioeconomic status* was assessed in terms of education and household income. Education was grouped into four categories: illiterate, only read and write, finished primary school, and completed junior high school and above. “Only read and write” were those who were not illiterate, but had not finish primary school. Household income per capita was adjusted for household size, yielding three categories: low, middle and high income according to the 25 and 75 percentiles.

*Child health* was also controlled in this study, since childhood health may have a consistent effect on later health outcomes. It was assessed retrospectively using the following questions: “how would you evaluate your health during childhood, up to and including age 15?” The choice comprised excellent, very good, good, fair, and poor. Though self-reported health in childhood may be subject to recall bias and measurement error, previous studies had confirmed that this measurement provides a good summary of overall childhood health [30, 31]. Researchers also found a strong correlation between self-reported health and many childhood physical diseases such as diabetes, respiratory diseases, heart diseases and ear infections [31]. Following Manor, we dichotomized child health into two categories: good (excellent, very good, good) and less than good (fair, poor) [32].

*Adult health and behaviors* were measured by responses to whether they had been diagnosed with a range of chronic diseases, including hypertension, diabetes, cancer, chronic lung diseases and asthma. The health behaviors examined whether respondents were currently smoking (yes or no) and currently drinking (yes or no).

*Other control variables* included ethnicity and marital status. Ethnicity was classified as Han and Minority; marital status was divided into two categories: married and others, which included divorced, widowed and never married.

### Ethical approval

CHARLS was approved by the Ethical Review Committee of Peking University and all participants signed informed consent at the time of participation.

### Statistical analysis

The relationship between socioeconomic indicators, health factors and functional disability was assessed using chi-square tests. Gender and socioeconomic-specific analyses were carried out to determine whether the associations between such exposures and functional disability differed by gender and socioeconomic group. Next, four stepwise binary logistic regressions were run for both ADL and IADL. The dependent variable in each model was a dichotomous variable, valued 1 if the respondents had functional disability; otherwise it took a value of 0. In model 1, individual characteristics were controlled. Model 2 further adjusted for childhood SES and health. Model 3 subsequently adjusted for adult SES and model 4 adjusted for chronic diseases and health behaviors. Odds ratios (OR), as well as 95% confidence intervals (CI), were used to compare the effect on the response variable. The level of significance was defined as 2-sided *p* value <0.05. All analyses were conducted using SPSS 19.0.

## Results

### Gender difference of socioeconomic and health factors

Table 1 lists the distribution of socioeconomic and health factors by gender. Overall, there were no differences between men and women in childhood SES. Roughly 90% of respondents were born in rural areas. For men, 36.0% of the respondents’ fathers had no education and for women, 35.1% of their father had no education. Most respondents (69%) reported their fathers’ main occupation as manual workers. As adults, women respondents experienced more disadvantages than men. As shown in Table 1, women were less educated, economically poorer and more likely to have chronic diseases than men. Health behavior differences were also found, with men more likely to be smokers and drinkers than women (*p* < 0.001).

**Table 1:** Gender difference of socioeconomic and health factors in Chinese older adults, 2013 (*n* = 18,448)

Variables	Men *n* (%)	Women *n* (%)	*P*-value
Childhood SES			
Birth place:			
Urban	923 (10.5)	947 (9.8)	0.118
Rural	7871 (89.5)	8707 (90.2)	
Father’s education:			
No education	4931 (56.1)	5465 (56.6)	0.271
Some education	3166 (36.0)	3388 (35.1)	
Father’s occupation:			
No manual	1913 (21.7)	2070 (21.5)	0.570
Manual	6068 (69.0)	6702 (69.4)	
Childhood health			
Good	6164 (70.1)	6652 (68.9)	0.005^**^
Less than good	1973 (22.4)	2348 (24.3)	
Adult SES			
Education:			
Illiterate	1097 (12.5)	3741 (38.7)	<0.001^**^
Only read and write	1581 (18.0)	1710 (17.7)	
Finished primary	2276 (25.9)	1764 (18.3)	
Junior high and above	3840 (43.6)	2439 (25.3)	
Household incomes per capita:			
Low	1918 (21.8)	2710 (28.1)	<0.001^**^
Middle	4301 (48.9)	4914 (50.9)	
High	2575 (29.3)	2030 (21.0)	
Adult health and behaviors			
Chronic diseases:			
No	3208 (36.5)	3218 (33.3)	<0.001^**^
Yes	5586 (63.5)	6436 (66.7)	
Currently smokers:			
No	6163 (70.1)	9292 (96.2)	<0.001^**^
Yes	2631 (29.9)	362 (3.8)	
Currently drinkers:			
No	4729 (53.8)	8877 (92.0)	<0.001^**^
Yes	4065 (46.2)	777 (8.0)	

^**^ *P* < 0.01

### Prevalence of ADL and IADL disabilities

Table 2 lists the prevalence of ADL and IADL disabilities. Overall, 19.5% of the women and 14.8% of the men reported ADL disabilities and 30.2% of the women and 20.5% of the men reported IADL disabilities. The overall prevalence of functional disability increased with advancing age, with the prevalence of ADL disability increasing from 10.6% in those aged 45–50 years to 43.7% of those aged 80 years and over. Respondents who were married reported the lowest prevalence in functional disability (*p* < 0.001). Those who were Minorities had more difficulties in ADL and IADL than Han.

**Table 2:** Prevalence of ADL and IADL disabilities in Chinese older adults, 2013 (*n* = 18,448)

Variables	*n* (%)	One or more difficulties in ADL *n* (%)	*P*-value	One or more difficulties in IADL *n* (%)	*P*-value
Gender:					
Men	8794 (47.7)	1304 (14.8)	<0.001^**^	1804 (20.5)	<0.001^**^
Women	9654 (52.3)	1878 (19.5)		2915 (30.2)	
Age group (years):					
45–59	9590 (52.0)	1013 (10.6)	<0.001^**^	1485 (15.5)	<0.001^**^
60–69	5485 (29.7)	1047 (19.1)		1587 (29.0)	
70–79	2552 (13.8)	761 (29.9)		1116 (43.9)	
≧80	821 (4.5)	355 (43.7)		517 (63.7)	
Marital status:					
Married	16,023 (86.9)	2462 (15.4)	<0.001^**^	3671 (22.9)	<0.001^**^
Other	2425 (13.1)	720 (29.7)		1048 (43.2)	
Ethnicity:					
Han	17,033 (92.3)	2908 (17.1)	0.028^*^	4297 (25.2)	<0.001^**^
Minority	1415 (7.7)	274 (19.4)		422 (29.8)	
Childhood SES					
Birth place:					
Urban	1870 (10.1)	234 (12.6)	<0.001^**^	270 (14.5)	<0.001^**^
Rural	16,578 (89.9)	2944 (17.8)		4447 (26.8)	
Father’s education:					
No education	10,396 (56.4)	2051 (19.7)	<0.001^**^	3169 (30.5)	<0.001^**^
Some education	6554 (35.5)	866 (13.2)		1173 (17.9)	
Father’s occupation:					
No manual	3983 (21.6)	575 (14.4)	<0.001^**^	724 (18.2)	<0.001^**^
Manual	12,770 (69.2)	2338 (18.3)		3569 (27.9)	
Childhood health					
Good	12,816 (69.5)	2127 (16.6)	0.006^**^	3175 (24.8)	0.020^*^
Less than good	4321 (23.4)	796 (18.4)		1147 (26.6)	
Adult SES					
Education:					
Illiterate	4838 (26.2)	1229 (25.4)	<0.001^**^	2210 (45.7)	<0.001^**^
Only read and write	3291 (17.8)	660 (20.1)		962 (29.2)	
Finished primary	4040 (21.9)	679 (16.8)		834 (20.6)	
Junior high and above	6279 (34.1)	614 (9.8)		713 (11.4)	
Household incomes per capita					
Low	4628 (25.1)	957 (20.9)	<0.001^**^	1450 (31.7)	<0.001^**^
Middle	9215 (50.0)	1749 (19.1)		2658 (29.0)	
High	4605 (24.9)	463 (10.2)		586 (12.9)	
Adult health and behaviors					
Chronic diseases:					
No	6426 (34.8)	542 (17.0)	<0.001^**^	8386 (61.1)	<0.001^**^
Yes	12,022 (65.2)	5884 (38.5)		3636 (77.1)	
Currently smokers:					
No	15,455 (83.8)	2771 (17.9)	<0.001^**^	4088 (26.5)	<0.001^**^
Yes	2993 (16.2)	411 (13.7)		631 (21.1)	
Currently drinkers:					
No	13,606 (73.8)	2605 (19.1)	<0.001^**^	3859 (28.4)	<0.001^**^
Yes	4842 (26.2)	577 (11.9)		860 (17.8)	

^**^ *P* < 0.01

For each socioeconomic indicator, respondents with disadvantaged SES background reported more ADL and IADL difficulties. In Table 2, low childhood SES, measured by rural birthplace, fathers’ low education and fathers’ manual occupation, were significantly related to functional disabilities. For adult SES, lower education and disadvantaged economic status were also associated with higher risk of functional disabilities (*p* < 0.001). Chronic diseases and health behaviors were related to both ADL and IADL disabilities (*p* < 0.001). Respondents with chronic diseases, non-smokers and non-drinkers, had more risk of functional disabilities.

### Logistic regressions for difficulties in ADL

Multiple logistic regressions of ADL disability are presented in Table 3. The individual characteristics based on model 1 showed that women, advancing age, unmarried were associated with ADL disability. After controlling for childhood SES and child health in Model 2, very few changes were observed in the gender odds (1.413 in Model 1 versus 1.373 in Model 2). Respondents whose father had no education had higher risks of ADL disabilities than those whose father was educated (OR = 1.264, 95% CI =1.111–1.437).

**Table 3:** Logistic regressions for difficulties in ADL, 2013

Variables	Model 1 (individual characteristics)	Model 2 (model 1+ childhood SES + child health)	Model 3 (model 2 + adult SES)	Model 4 (model 3 + adult health)
	OR (95% CI); *p-value*	OR (95% CI); *p-value*	OR (95% CI); *p-value*	OR (95% CI); *p-value*
Individual characteristics				
Gender: women (vs men)	1.413 (1.287–1.551); <0.001^**^	1.373 (1.239–1.521); <0.001^**^	1.191 (1.065–1.332); 0.002^**^	1.058 (0.929–1.206);0.392
Age group:				
60–69 (vs <45–59)	2.131 (1.889–2.405); <0.001^**^	1.949 (1.718–2.211); <0.001^**^	1.696 (1.491–1.930); <0.001^**^	1.513 (1.331–1.720); <0.001^**^
70–790	3.697 (3.210–4.257); <0.001^**^	3.331 (2.857–3.884); <0.001^**^	2.871 (2.432–3.388); <0.001^**^	2.518 (2.141–2.961); <0.001^**^
≧80	6.554 (5.239–8.200); <0.001^**^	5.243 (3.980–6.907); <0.001^**^	4.333 (3.335–5.628); <0.001^**^	3.910 (3.008–5.082); <0.001^**^
Marital status: not married (others)	1.390 (1.200–1.611); <0.001^**^	1.365 (1.165–1.601); <0.001^**^	1.349 (1.159–1.570); <0.001^**^	1.372 (1.180–1.596); <0.001^**^
Ethnicity: Minority (vs Han)	1.243 (0.978–1.581); 0.076	1.218 (0.918–1.616); 0.171	1.167 (0.884–1.541); 0.274	1.112 (0.860–1.438); 0.419
Childhood SES				
Birth place: rural (vs urban)		1.272 (0.987–1.638); 0.063	1.020 (0.789–1.319); 0.879	1.018 (0.786–1.319); 0.893
Father’ education: no education (vs some education)		1.264 (1.111–1.437); <0.001^**^	1.138 (0.998–1.298); 0.054	1.144 (0.999–1.310); 0.053
Father’s occupation: Manual (vs non-manual)		0.999 (0.854–1.168); 0.985	0.892 (0.767–1.038); 0.140	0.917 (0.782–1.075); 0.284
*Childhood health:* less than good (vs good)		1.042 (0.903–1.204); 0.570	1.051 (0.919–1.203); 0.466	1.017 (0.883–1.171); 0.817
Adult SES				
Education (vs junior high and above)				
Illiterate			1.634 (1.314–2.030); <0.001^**^	1.682 (1.350–2.095); <0.001^**^
Only read and write			1.552 (1.273–1.893); <0.001^**^	1.567 (1.283–1.913); <0.001^**^
Finished primary			1.426 (1.177–1.728); <0.001^**^	1.428 (1.180–1.729); <0.001^**^
Household incomes per capita:				
Low (vs high)			1.831 (1.532–2.188); <0.001^**^	1.852 (1.537–2.232); <0.001^**^
Middle (vs high)			1.624 (1.355–1.947); <0.001^**^	1.583 (1.313–1.907); <0.001^**^
Adult health and behaviors				
Chronic diseases: yes (vs no)				2.792 (2.418–3.224); <0.001^**^
Currently smokers: no (vs yes)				1.126 (0.932–1.359); 0.218
Currently drinkers: no (vs yes)				1.279 (1.094–1.494); 0.002^**^

^**^ *P* < 0.01

Model 3 further adjusted for adult SES, where gender, advancing age, marital status were also associated with functional disability. Fathers’ education in Model 3 consistently predicted ADL disability, but the association was not significant. Adult low SES, poor education and low household income per capita were significant predictors of ADL disability, even after childhood conditions were controlled.

Model 4 subsequently controlled adult health and health behaviors. The gender difference disappeared, but advancing age and marital status remained significantly related to ADL disability. The association between lower childhood SES and ADL disability was not significant. Poor SES in adulthood predicted the higher risk of ADL disability as did respondents with chronic diseases (OR = 2.792, 95% CI = 2.418–3.224). Contrary to our expectations, those who were non-drinkers had higher odds ADL disabilities (OR = 1.279, 95% CI = 1.094–1.494).

### Logistic regressions for difficulties in IADL

Model 1 in Table 4 showed that gender, age, marital status and nationality were associated with the risk of IADL disabilities. Controlling for childhood conditions, Model 2 showed that respondents born in rural areas and fathers with no education and manual occupations had increased risks of IADL disabilities.

**Table 4:** Logistic regressions for difficulties in IADL, 2013

Variables	Model 1 (individual characteristics)	Model 2 (model 1+ childhood SES + child health)	Model 3 (model 2 + adult SES)	Model 4 (model 3 + adult health)
	OR (95% CI); *p-value*	OR (95% CI); *p-value*	OR (95% CI); *p-value*	OR (95% CI); *p-value*
Individual characteristics				
Gender: women (vs men)	1.739 (1.588–1.904); <0.001^**^	1.655 (1.505–1.821); <0.001^**^	1.185 (1.067–1.314); <0.002^**^	1.091 (0.966–1.233); 0.167
Age group:				
60–69 (vs <45–59)	2.350 (2.074–2.663); <0.001^**^	2.111 (1.853–2.406); <0.001^**^	1.685 (1.482–1.915); <0.001^**^	1.567 (1.378–1.781); <0.001^**^
70–79	4.429 (3.859–5.083); <0.001^**^	4.002 (3.462–4.626); <0.001^**^	3.058 (2.618–3.572); <0.001^**^	2.808 (2.406–3.278); <0.001^**^
≧80	9.639 (7.398–12.558); <0.001^**^	7.310 (5.306–10.071); <0.001^**^	5.037 (3.850–6.590); <0.001^**^	4.687 (3.582–6.134); <0.001^**^
Marital status: not in marriage (in marriage)	1.485 (1.268–1.739); <0.001^**^	1.454 (1.228–1.723); <0.001^**^	1.370 (1.168–1.607); <0.001^**^	1.380 (1.177–1.618); <0.001^**^
Ethnicity: Minority (vs Han)	1.450 (1.115–1.887); <0.006^**^	1.385 (1.059–1.811); 0.02^*^	1.278 (0.993–1.645); 0.057	1.239 (0.978–1.568); 0.075
Childhood SES				
Birth place: rural (vs urban)		1.688 (1.336–2.133); <0.001^**^	1.157 (0.912–1.468); 0.229	1.154 (0.910–1.463); 0.236
Father’s education: no education (vs some education)		1.482 (1.321–1.663); <0.001^**^	1.194 (1.060–1.345); 0.004^**^	1.198 (1.062–1.353); 0.004^**^
Father ‘s occupation: manual (vs no manual)		1.258 (1.082–1.462); 0.003^**^	1.050 (0.907–1.214); 0.514	1.071 (0.923–1.243); 0.363
*Childhood health:* less than good (vs good)		1.025 (0.892–1.178); 0.729	1.032 (0.914–1.166); 0.608	1.010 (0.890–1.146); 0.878
Adult SES				
Education (vs junior high and above)				
Illiterate			3.218 (2.660–3.893); <0.001^**^	3.300 (2.721–4.000); <0.001^**^
Only read and write			2.114 (1.762–2.536); <0.001^**^	2.133 (1.776–2.561); <0.001^**^
Finished primary			1.483 (1.259–1.746); <0.001^**^	1.483 (1.261–1.743); <0.001^**^
Household incomes per capita:				
Low (vs high)			2.108 (1.764–2.520); <0.001^**^	2.118 (1.761–2.547); <0.001^**^
Middle (vs high)			1.826 (1.535–2.173); <0.001^**^	1.793 (1.502–2.141); <0.001^**^
Adult health and behaviors				
Chronic diseases: yes (vs no)				1.842 (1.652–2.053); <0.001^**^
Currently smokers: no (vs yes)				1.047 (0.886–1.236); 0.590
Currently drinkers: no (vs yes)				1.203 (1.053–1.374); 0.006^**^

^**^ *P* < 0.01

Model 3 showed that after controlling for adult SES, the relationship between birthplace, father’s occupation and IADL disabilities disappeared. Father’s education consistently predicted IADL disabilities, and the respondents whose father had no education had an increased risk of IADL disabilities (OR = 1.194, 95% CI =1.060–1.345). As for adult SES, respondents with lower than junior high education were significantly related to high rates of IADL disabilities, as were those with lower and middle household income.

Model 4 further controlled adult health and health behaviors. Having chronic diseases meant a higher rate of IADL disabilities (OR = 1.842, 95% CI = 1.652–2.053). Similar to ADL disabilities, respondents who were not drinkers had, surprisingly, higher odds IADL disabilities (OR = 1.203, 95% CI = 1.053–1.374).

## Discussion

### Main findings

Using national representative data from CHARLS, we tested the impact of gender and childhood and adult SES on functional disability of older Chinese adults. There was no gender difference after adult conditions were controlled, while SES was confirmed as a significant factor in determining functional disability. The results showed that harsh SES in childhood, especially those with fathers with no education, was an important predictor of functional disability. Adult socioeconomic inequalities explained functional disability: a poor education and low income were found to be associated with functional disability. In addition, advancing age, not being married, having chronic diseases, and non-drinkers were also related to functional disability.

### Gender difference in functional disability

In our study, gender difference disappeared after controlling for adult conditions. Though there existed differences in socioeconomic and health conditions between women and men, it did not explain gender difference in the prevalence of functional disability. While a Singaporean study found women had higher prevalence in difficulties with all the basic activities of living than men, the research employed only basic analyses and did not control for socioeconomic conditions [8]. A study from England showed men had higher risk of functional disability, but it measured functional disability by indicators including hearing loss instead of traditional instruments such as ADL and IADL [9]. Our results are consistent with Rodrigues’ review [4] of 21 disability studies and Hu’s research [16] on older Chinese that gender was not a determiner of functional disability. To reduce functional disability in men and women, policy measures should act on the risk factors amenable to intervention, such as socioeconomic inequalities, regardless of gender.

### Socioeconomic inequalities in functional disability

All indicators of childhood SES were related to functional disability, but in our multivariate analyses only father’s education on IADL was a predictive factor. Previous studies have shown that in adulthood, children of parents’ with a higher level of education, had low levels of functional disability [33]. Parents’ higher education can influence healthier child eating habits, more physical exercise and better mental health, which promotes higher functioning [34, 35]. In our study, father’s education was related to IADL, but not ADL, which means childhood skills from educated households’ improved higher level tasks, such as, grocery shopping and managing money, in old age. Since childhood is a critical period during one’s development, we expected to find that childhood SES limited access to social resources and material well-being, which are vital for children’s adult health outcomes.

We did find that disadvantages in adult education and household income contributed to both ADL and IADL disabilities. Lack of education is often related to lower standards of living, more chronic diseases and less frequent use of health care services, which helps explain functional disabilities in older Chinese adults. As previous studies have reported [11], insufficient income was a strong predictor of functional disabilities. Income has been consistently associated with health outcomes, influencing not only exposure to health risk factors, but also access to resources that allows older Chinese to modify their current living conditions. Low income in older adults also hinders access to health service and social services, forming part of the causal chain linking income levels, health factors and functional disabilities [36, 37].

As discussed above, our results indicated that after adjusting for adult SES, the association between childhood SES and disabilities was attenuated. From the life course perspectives linking childhood SES to later life outcomes, the pathway model emphasized the impact of early SES conditions on life course trajectories and later life outcomes. However, disadvantages associated with poor backgrounds in childhood may be mediated by later SES attainment, such as educational level and income [38]. When adult SES was controlled in our regression models, the effect of childhood SES on functional disability was decreased, which suggests that improved adult SES reduce the negative childhood SES impacts. But, even if current SES was a better predictor of health than conditions of origin, Harper [39] argued that early social conditions shaped later social status attainment and then health. Further, China’s record of poverty reduction and the rising urbanization rate over the past 30 years likely attenuated childhood SES disadvantages as a factor affecting functional disability. We believe that directing public policy attention to improving childhood SES will pay dividends on health outcomes decades later as will improvements to adult SES that also bring health outcome benefits in old age.

### Other factors and functional disability

Consistent with previous studies, chronic diseases was an important factor determining functional disabilities [4, 15]. Living with chronic diseases and ADL/IADL disabilities is a burden for the elderly and their caregivers. This finding is important for targeting appropriate prevention and intervention strategies. Programs and policies that promote health should target older adults with chronic diseases. The strategy includes developing comprehensive service systems covering preventive, medical, physical, psychological and environmental aspects [40]. Another finding in this study was that those who were not drinkers had higher risk of functional disability, both in ADL and IADL. Consistent with previous studies [41], consuming some alcohol may improve health outcomes compared to non-drinkers.

Our study also showed that advanced age is strongly associated with functional disability [15]. Age-related functional disabilities impose substantial demands for health-care and significant costs on China’s health system. As the population ages, people with functional disabilities are projected to increase significantly. Policy interventions need to address the multiple causes of functional disability, including improving childhood and adult SES, disease prevention and early health care interventions, to contain growth health care costs.

### Limitations

The study has several limitations. First, childhood conditions including SES and health were obtained by retrospective self-report. Previous studies have shown that for objective measures, such as parental education and occupation, retrospective reports tend to be fairly reliable [38], although we acknowledge potential recall bias. For childhood health, Smith demonstrated that child health proxied by childhood physical diseases was a good proxy for childhood health [30]. Second, the study has been careful not to claim a causal relationship between childhood SES, child health and later functional disability based on cross-sectional data. But future research needs to investigate such causal relationships.

## Conclusions

The prevalence of functional disability in older Chinese adults is high, especially for those with chronic diseases and from disadvantaged SES backgrounds. Our findings suggest that the “long arm” of childhood SES indirectly impacts functional disability at older ages, while adult SES impacts disability more directly. Policies that initially benefit children will yield well-being benefits as men and women reach adulthood. Promoting ongoing childhood and adult SES advantages reduce the likelihood that older adults will suffer from functional disability. While there is better access to education and higher income levels for Chinese children today, both children and adults suffer from significant income inequality and urban-rural differentials in income and access to health facilities [42, 43]. Policies to reduce social inequalities should be multi-sectorial and based on promoting equality from early life to old age.

## Abbreviations

ADL: Activities of daily life
CHARLS: China Health and Retirement Longitudinal Study
IADL: Instrumental activities of daily life
SES: Socioeconomic status
